# Supplementary material for: The STRATAA study protocol: a programme to assess the burden of enteric fever in Bangladesh, Malawi and Nepal using prospective population census, passive surveillance, serological studies and healthcare utilisation surveys
Source: BMJ Open. 2017 Jul 2;7(6):e016283. doi: 10.1136/bmjopen-2017-016283 (PMC5726077; doi:10.1136/bmjopen-2017-016283)
Supplement: Supplementary data [file bmjopen-2017-016283supp001.pdf]

Supplemental Table 1. Overview of STRATAA Study Procedures.

**Study component**	**CENSUS**							**HEALTHCARE UTILISATION**		**PASSIVE SURVEILLANCE**									**SEROSURVEY**			
								Peak seasons during census		All cases					Household contacts of index cases				Participants			Household contacts of possible carriers
										Cases within census area*												
Sample size (individuals unless specified)	≥100 000 per site							>700 households		Convenience					≥73 households				8500		6800	Members of ≥50 households
**Time (months, unless specified)**	**0**	**4**	**8**	**12**	**16**	**20**	**24**	**1st**	**2nd**	**0**	**Day 8**	**1**	**~6**	**12**	**0**	**1**	**~3**	**~12**	**0**	**	**3**	**6**
Consent	X							X		X					X		X[§$]		X			x
Demographic Information	X	X[#]	X[#]	X[#]	X[#]	X[#]	X															
Clinical information										X	X	X	X	X	X							
Questionnaire								X	X													
Blood sample										X[@]			X		X		X[$]		X[@]		X	X
Stool sample										X		X	X	(X)	X	X	2X[€]	(3X)		2X[€]		X
Urine sample										X[@]									X[@]			

Supplemental Table 1. Overview of STRATAA Study Procedures.

* Case within census area = index if blood-culture confirmed typhoid diagnosis, resident in census area and household members consent to participation.

\# Births, deaths and migrations updated every 4 months in Nepal & Bangladesh

~ approximately - dependent on timeframe of availability for the Vi ELISA result

$ Host genetics collected/used for laboratory studies if either stool or serum positive or both

§ separate consent for host genetics

@ additional blood (RNA + plasma) and urine samples (subset of 100 with typhoid (culture positive), 100 with clinically suspected typhoid (culture negative), 100 with alternate bacteraemia; and 100 healthy controls (from the serosurvey) from adults 16-40yrs who give consent for genetic assays to be performed.

€ Participants identified as having high anti-Vi IgG only - 2 stool samples collected 48 hours apart once ELISA result known to identify/confirm carriage

** When result known

Total blood volumes: **Passive survey** ≤16 mL adults (>16 years), ≤7 mL children (<16 years); **Serosurvey** ≤8 mL adults (>16 years), ≤7 mL children. Cell pellets stored for host genetics.
